# Preliminary *In Silico* Evaluation of Extra Virgin Olive Oil-Derived Bioactive Compounds as Multi-Target-Directed Ligands in Metabolic Dysfunction-Associated Steatotic Liver Disease

**DOI:** 10.3390/life16071146

**Published:** 2026-07-10

**Authors:** Ludovico Abenavoli, Maja Milanović, Giuseppe Guido Maria Scarlata, Nataša Milošević, Maria Luisa Gambardella, Nataša Milić

**Affiliations:** 1 Department of Health Sciences, University “Magna Graecia”, 88100 Catanzaro, Italy; giuseppeguidomaria.scarlata@unicz.it (G.G.M.S.); marialuisa.gambardella@studenti.unicz.it (M.L.G.); 2 Center for Chronic Liver Diseases, “Renato Dulbecco” University Hospital, 88100 Catanzaro, Italy; 3 Department of Pharmacy, Faculty of Medicine, University of Novi Sad, Hajduk Veljkova 3, 21000 Novi Sad, Serbia; maja.milanovic@mf.uns.ac.rs (M.M.); natasa.milosevic@mf.uns.ac.rs (N.M.); natasa.milic@mf.uns.ac.rs (N.M.)

**Keywords:** oleuropein, polyphenols, PPAR signaling, molecular docking, mediterranean diet, nutraceuticals

## Abstract

**Background:** Metabolic dysfunction-associated steatotic liver disease (MASLD) is the most prevalent chronic liver disease worldwide and is driven by complex metabolic and inflammatory disturbances. Extra virgin olive oil (EVOO), a hallmark of the Mediterranean diet, contains numerous bioactive compounds that may exert beneficial effects on liver and cardiometabolic health. This preliminary study investigated the interactions of selected EVOO-derived compounds, with molecular targets implicated in MASLD using an integrated in silico approach. **Methods:** Phenolic compounds, secoiridoids, fatty acids, sterols, squalene, and vitamin E were evaluated. Physicochemical properties, drug-likeness, and pharmacokinetic profiles were predicted using ADMETlab 3.0. Molecular docking analyses were performed against liver X receptors (LXRα and LXRβ), peroxisome proliferator-activated receptors (PPARα and PPARγ), hydroxymethylglutaryl-CoA reductase, cyclooxygenase-1, and cyclooxygenase-2. Binding modes were further examined by three-dimensional interaction analyses. **Results:** The investigated compounds displayed heterogeneous physicochemical and pharmacokinetic profiles. Oleuropein, oleacein, and oleocanthal demonstrated the most consistent binding patterns across targets involved in lipid metabolism, inflammation, and cardiometabolic regulation. In contrast, highly lipophilic compounds, including squalene, β-sitosterol, and vitamin E, frequently achieved high docking scores but formed fewer biologically relevant interactions. **Conclusions:** EVOO phenolics, particularly oleuropein, oleacein, and oleocanthal, emerged as promising multi-target modulators of MASLD-related pathways, supporting the potential role of EVOO in MASLD prevention and management.

## 1. Introduction

Metabolic dysfunction-associated steatotic liver disease (MASLD) is currently the most prevalent chronic liver disease worldwide, affecting approximately 38% of the adult population and posing a major global health burden [1]. MASLD encompasses a spectrum of progressive liver disorders, ranging from simple steatosis to metabolic dysfunction-associated steatohepatitis, fibrosis, cirrhosis, and hepatocellular carcinoma [2]. Despite its increasing prevalence and substantial clinical impact, effective pharmacological treatment options remain limited, and lifestyle interventions continue to represent the cornerstone of disease prevention and management [3].

Among lifestyle-based intervention, the Mediterranean diet has emerged as one of the most effective nutritional strategies for patients with MASLD. Characterized by a high consumption of plant-derived foods, monounsaturated fatty acids, and bioactive compounds, this dietary pattern has consistently been associated with improvements in metabolic health and reduced cardiovascular risk; conditions that frequently coexist with MASLD [4,5]. Recent clinical evidence suggests that greater adherence to the Mediterranean diet may reduce the risk of advanced liver fibrosis and improved metabolic outcomes in affected individuals [6].

Extra virgin olive oil (EVOO), the principal source of dietary fat in the Mediterranean diet, is considered one of the major contributors to these beneficial effects. Unlike refined olive oils, EVOO retains a complex phenolic fraction rich in hydroxytyrosol, tyrosol, oleuropein, oleocanthal, oleacein, and related secoiridoids [7,8,9]. Experimental and clinical data suggest that these bioactive compounds exert antioxidant, anti-inflammatory, lipid-modulating, and metabolic regulatory effects, potentially influencing multiple molecular pathways involved in MASLD pathogenesis [10].

The pathogenesis of MASLD is highly complex and multifactorial, involving dysregulated lipid metabolism, oxidative stress, chronic inflammation, and progressive fibrogenesis. Accordingly, several EVOO-derived polyphenols have been shown to modulate key molecular pathways implicated in disease progression, including nuclear receptor signaling, inflammatory mediators, and metabolic regulators [11,12,13,14]. Their pleiotropic biological activity suggests that these compounds may act as multi-target-directed ligands, simultaneously modulating multiple interconnected pathogenic mechanisms. Although increasing evidence supports the beneficial role of EVOO and its phenolic constituents in MASLD, comparative investigations exploring their interactions with multiple molecular targets remain scarce [10].

Therefore, the aim of this preliminary study was to evaluate, through an integrated in silico approach combining molecular and physicochemical profiling and molecular docking analysis, the interactions of selected EVOO-derived compounds with key targets involved in MASLD pathogenesis, with the objective of identifying potential multi-target-directed ligands.

## 2. Materials and Methods

### 2.1. Ligand Library Preparation

A library of bioactive compounds representative of EVOO was compiled based on the published literature. The selected molecules included phenolic compounds (hydroxytyrosol, tyrosol, and their derivatives), secoiridoids (oleuropein, oleuropein aglycone, oleacein, oleocanthal), fatty acids, sterols, and highly lipophilic molecules such as squalene and vitamin E. SMILES strings of the selected compounds were retrieved from the PubChem database and converted into 2D structures using ChemDraw Professional 16.0.

**Figure 1:**
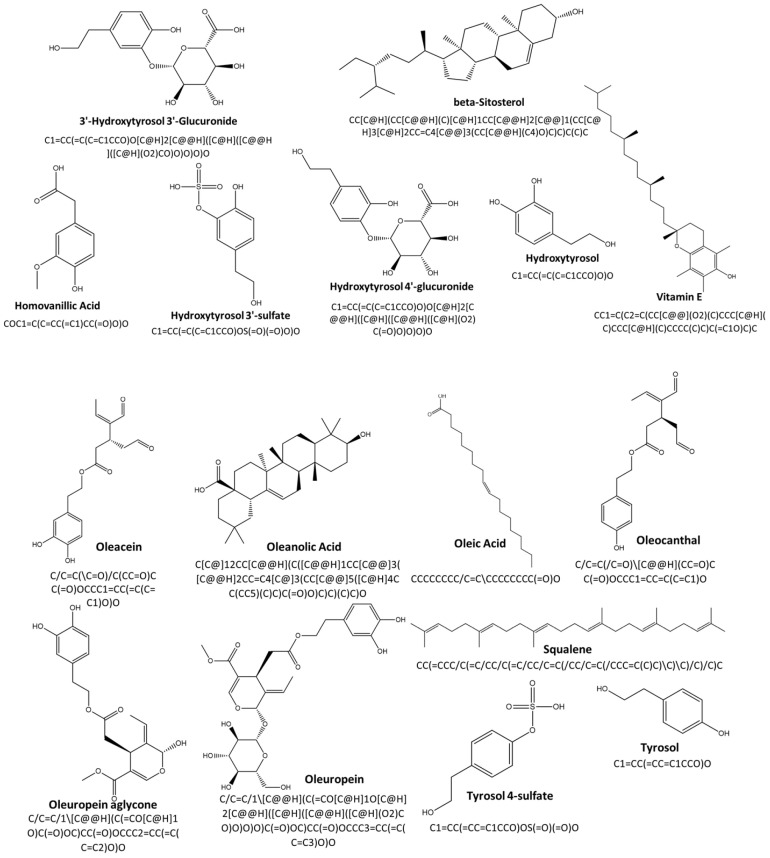
Chemical structures of EVOO-derived compounds analyzed. SMILES notation is reported for each molecule.

The chemical structures and corresponding SMILES strings of the investigated compounds are presented in Figure 1.

### 2.2. Molecular Properties and Bioavailability Profiles

Molecular descriptors and physicochemical properties were predicted in silico using ADMETlab 3.0 (https://admetlab3.scbdd.com/ (accessed on 5 April 2026)). The evaluated parameters included molecular weight (M_W_), volume (V), number of rings (nRing), number of rotatable bonds (nRot), hydrogen-bond donors and acceptors (nHD and nHA), topological polar surface area (TPSA), and fraction of sp^3^ carbons (Fsp^3^).

Lipophilicity (logP and logD7.4), aqueous solubility (logS), human intestinal absorption (HIA), and blood–brain barrier (BBB) permeability were also assessed to estimate drug-likeness and oral bioavailability profile. According to Lipinski’s rule of five, compounds with good oral bioavailability generally violate no more than one of the following criteria: M_W_ < 500 Da, nHD ≤ 5, nHA ≤ 10 and logP < 5 [15]. According to Veber’s criteria, favorable oral bioavailability is expected for compounds with nRot ≤ 10 and TPSA ≤ 140 Å^2^ [16].

### 2.3. Target Selection and Preparation

Based on their established roles in lipid metabolism, inflammation, and MASLD pathogenesis, in accordance with previously validated multi-target approaches [17], the following targets were selected: liver X receptor alpha (LXRα), liver X receptor beta (LXRβ), peroxisome proliferator-activated receptor alpha (PPARα) and gamma (PPARγ), along with the enzymes hydroxymethylglutaryl-CoA reductase (HMG-CoA reductase), cyclooxygenase-1 (COX-1) and cyclooxygenase-2 (COX-2).

The 3D crystal structures of the selected targets in complex with co-crystallized ligands were retrieved from the RCSB Protein Data Bank (PDB, https://www.rcsb.org/ (accessed on 9 April 2026)) in PDB format (Table 1). The Protein Preparation Wizard incorporated in Genetic Optimisation for Ligand Docking (GOLD, version 2022.3.0) was used for target preparation. All water molecules and co-crystallized ligands were removed, to ensure a standardized preparation protocol, across all tested targets. Missing hydrogen atoms were added to the protein structures prior to docking simulations.

### 2.4. Docking Protocol and Validation

Molecular docking simulations were performed using a validated protocol in GOLD (version 2022.3.0) based on flexible ligand-rigid receptor docking [17]. The SMILES strings of the EVOO-derived compounds were converted into 3D structures using the Chem3D tool (version 16.0). The MMFF94 force field was applied for the ligand geometry optimization. The ligand-binding domain for each target was defined within a 6 Å radius around the coordinates of the co-crystallized ligand. The docking protocol was validating by redocking the co-crystallized ligands (e.g., T0901317, lanifibranor, GW1929, mevastatin, flurbiprofen, and naproxen) into their respective binding sites. The obtained RMSD values were 1.3404 for LXRα, 1.2824 for LXRβ, 0.8773 for PPARα, 0.7786 for PPARγ, 1.2193 for HMG-CoA reductase and 0.5038 and 0.4001 for COX-1 and COX-2, respectively. All values were within acceptable thresholds (RMSD < 2 Å) for each target, confirming accurate identification of the binding site and reproduction of key interactions [18,19]. For each EVOO-derived compound and each selected target, ten docking poses were generated using the ChemPLP scoring function. The corresponding ChemPLP scores were calculated. The pose with the highest ChemPLP score was selected for subsequent interaction analysis [8,17,18,19].

### 2.5. Binding-Mode Analysis

Protein–ligand interactions were analyzed, including hydrogen bonding, hydrophobic interactions, π–π interactions (where applicable), and van der Waals interactions. The academic version of Maestro Schrödinger software (v.14.7.) was used for visualization of the selected protein–ligand complexes.

## 3. Results

### 3.1. Molecular Properties and Bioavailability Profiles

The analyzed EVOO-derived compounds displayed considerable variability in their molecular properties (Table 2). Phenolic compounds, such as hydroxytyrosol and its conjugates, had low molecular weights and high polarity, whereas sterols and lipidic compounds, such as β-sitosterol, squalene, and vitamin E, were characterized by higher molecular weights, larger molecular volumes, and higher Fsp^3^ values. Oleuropein was the only polyphenolic compound that had more than 10 rotatable bonds. In addition, the high polarity of oleuropein and hydroxytyrosol glucuronides corresponded to the increased total number of hydrogen-bond donors and acceptors.

Marked differences in lipophilicity and predicted permeability were observed among the analyzed compounds (Table 3). Highly lipophilic compounds (logP/logD_7.4_ > 5), including squalene, β-sitosterol, and vitamin E, exhibited poor aqueous solubility and limited predicted intestinal absorption. In contrast, conjugated phenolic derivatives exhibited improved solubility but reduced membrane permeability. Among the studied EVOO compounds, only oleanolic acid and vitamin E exhibited permeation through the BBB, while predicted high human intestinal absorption was limited to 3′-hydroxytyrosol 3′-glucuronide, hydroxytyrosol 3′-sulfate, and tyrosol 4-sulfate. To complement the in silico predictions, Lipinski’s and Veber’s empirical rules were applied to further evaluate drug-likeness and oral bioavailability. Although the majority of compounds met criteria set by empirical rules, violations frequently occurred due to high lipophilicity (β-sitosterol, oleic acid, squalene, and vitamin E) and an elevated number of rotatable bonds (oleic acid, oleuropein, squalene, and vitamin E). Interestingly, oleuropein did not meet Lipinski’s and Veber’s criteria due to its high molecular weight (540.18), increased polarity (nHA = 13 and TPSA = 201.67 Å^2^), and 11 rotatable bonds. Among the other phenolic compounds, only hydroxytyrosol 4′-glucuronide exhibited more than one violation, driven by a high number of hydrogen-bond donors (nHD = 6) and a high TPSA (156.91 Å^2^).

### 3.2. Docking Performance and Score Interpretation

Docking simulations demonstrated heterogeneous ChemPLP scores across the analyzed targets. Since the ChemPLP score is a dimensionless scoring function and is based on an empirical fitness function optimized for pose prediction within a specific binding site, the obtained scores were interpreted relative to the scores of co-crystalized ligands within each individual target. Consequently, the docking results were used to rank compounds within each target rather than to compare binding affinities between targets with different pocket properties. (Table 4).

Within individual targets, several highly lipophilic compounds, particularly squalene, vitamin E, and β-sitosterol, achieved high docking scores, in some cases, comparable to those of reference ligands. However, squalene, vitamin E, β-sitosterol and oleic acid exhibited also very high values of lipophilicity (logP > 7, Table 3), which may lead to an overestimation of binding affinity in molecular docking analyses. In contrast, phenolic compounds and secoiridoids with logP values below 3 demonstrated more consistent ChemPLP scores across all examined targets in comparison with co-crystalized ligands.

### 3.3. Target-Specific Binding-Mode Analysis

Despite the high docking scores, several highly lipophilic compounds did not establish meaningful interactions within the ligand-binding domain of the target proteins, as evidenced by the absence of hydrogen bonds or stable contacts in the 3D representations.

**Figure 2:**
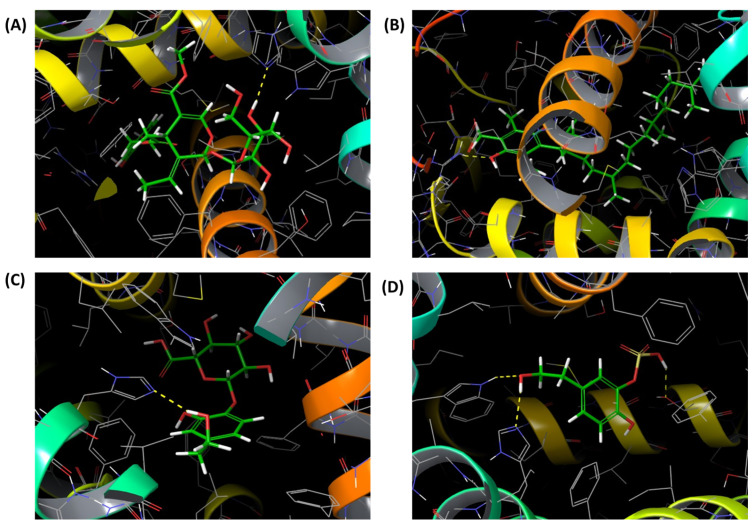
Three-dimensional representation of LXRα complexed with oleuropein (**A**), vitamin E (**B**), hydroxytyrosol 4′-glucuronide (**C**) and hydroxytyrosol 3′-sulfate (**D**). Yellow dash indicates hydrogen bonds.

Binding-mode analysis performed on LXRα indicated that oleuropein and vitamin E adopted stable conformations within the ligand-binding domain, forming hydrogen bonds. In contrast, β-sitosterol and squalene, despite high ChemPLP scores, did not establish meaningful interactions. Even the compounds that expressed lower ChemPLP scores—tyrosol, hydroxytyrosol, hydroxytyrosol 4′-glucuronide and hydroxytyrosol 3′-sulfate—like oleuropein, reproduced the hydrogen-bond interaction with His421 observed in co-crystalized ligand, T0901317 (Figure 2).

**Figure 3:**
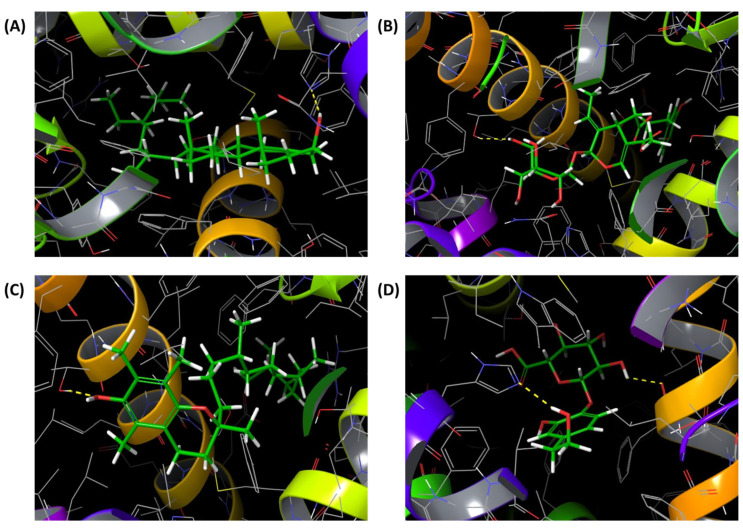
Three-dimensional representation of LXRβ complexed with β-sitosterol (**A**), oleuropein (**B**), vitamin E (**C**) and hydroxytyrosol 4′-glucuronide (**D**) Hydroxytyrosol 4′-glucuronide. Yellow dash indicates hydrogen bonds.

A similar interaction pattern was observed for LXRβ, where oleuropein, β-sitosterol and vitamin E formed stable hydrogen-bond networks within the ligand-binding domain, resulting in more favorable binding modes, while squalene failed to form stabilizing contacts (Figure 3). β-sitosterol reproduced the hydrogen bond with His435 as proven ligand, T0901317, whereas oleuropein and vitamin E formed polar interactions with Thr272. Interestingly, despite its lower ChemPLP values (65.929), oleocanthal established a dense hydrogen-bond network involving Thr272. Hydroxytyrosol 4′-glucuronide and hydroxytyrosol 3′-sulfate were stabilized with the ligand-binding pocket by creating multiple hydrogen bonds, most notably with His435, similar to β-sitosterol.

**Figure 4:**
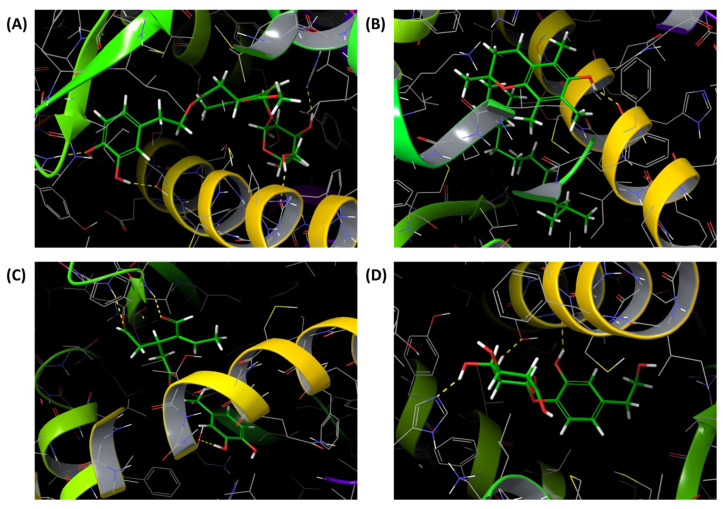
Three-dimensional representation of PPARα complexed with oleuropein (**A**), vitamin E (**B**), oleacein (**C**) and hydroxytyrosol 4′-glucuronide (**D**) Hydroxytyrosol 4′-glucuronide. Yellow dash indicates hydrogen bonds.

For PPARα, oleuropein and vitamin E indicated more consistent binding modes, whereas oleic acid and squalene did not establish any interactions, suggesting limited biological relevance. The stability of the oleuropein–PPARα complex was attributed to four hydrogen bonds associated with stable binding with PPARα. In addition, phenolic compounds like oleacein, hydroxytyrosol 4′-glucuronide and oleocanthal formed multiple hydrogen bonds within the PPARα pocket (Figure 4).

**Figure 5:**
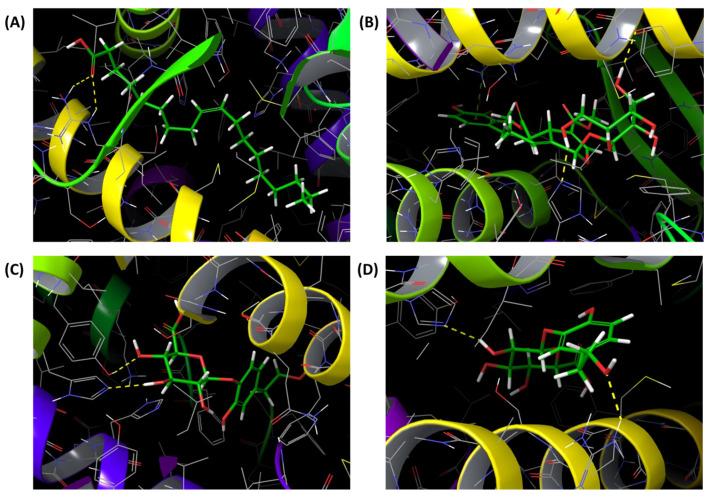
Three-dimensional representation of PPARγ complexed with oleic acid (**A**), oleuropein (**B**), hydroxytyrosol 4′-glucuronide (**C**), and 3′-hydroxytyrosol 3′-glucuronide (**D**). Yellow dash indicates hydrogen bonds.

For PPARγ, among the analyzed compounds, oleic acid, oleuropein, oleacein, and vitamin E demonstrated stable binding conformations within the ligand-binding domain (Figure 5). Again, hydrogen bonds were responsible for the stability of the created ligand–target complexes. Even the phenolic compound that exhibited lower ChemPLP scores similar to 3′-hydroxytyrosol 3′-glucuronide, hydroxytyrosol 4′-glucuronide and oleocanthal established multiple hydrogen interactions with the receptor.

**Figure 6:**
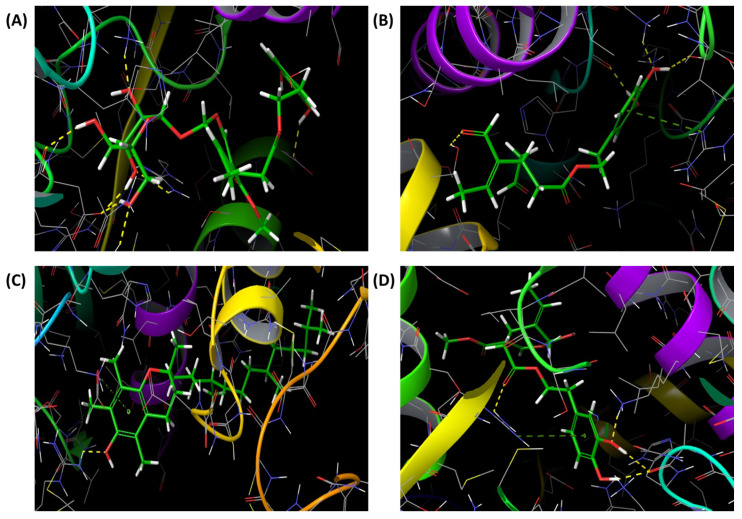
Three-dimensional representation of HMG-CoA reductase complexed with oleuropein (**A**), oleacein (**B**), vitamin E (**C**), and oleuropein aglycone (**D**). Yellow dash indicates hydrogen bonds while green dash is related to π–cation interaction.

About HMG-CoA reductase, docking analysis indicated that oleuropein, oleacein and vitamin E formed an extensive hydrogen-bond network within the catalytic site, whereas squalene, despite high scores, did not exhibit relevant binding interactions (Figure 6). Within the complex mainly hydrogen-bond-driven network, oleuropein, vitamin E, oleacein, and even oleuropein aglycone demonstrated interactions with Arg590, resembling the interaction pattern of the reference ligand mevastatin. In addition, oleuropein formed an additional hydrogen bond with the Asp690 amino-acid residue similar to mevastatin.

**Figure 7:**
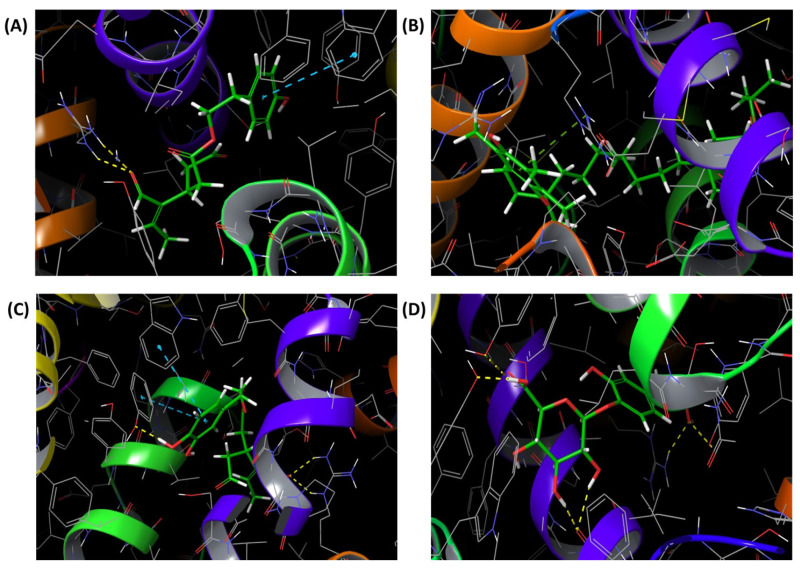
Three-dimensional representation of COX-1 complexed with oleocanthal (**A**), vitamin E (**B**), oleacein (**C**) and hydroxytyrosol 4′-glucuronide (**D**). Yellow dash indicates hydrogen bonds while blue dash is related to π-π interactions.

**Figure 8:**
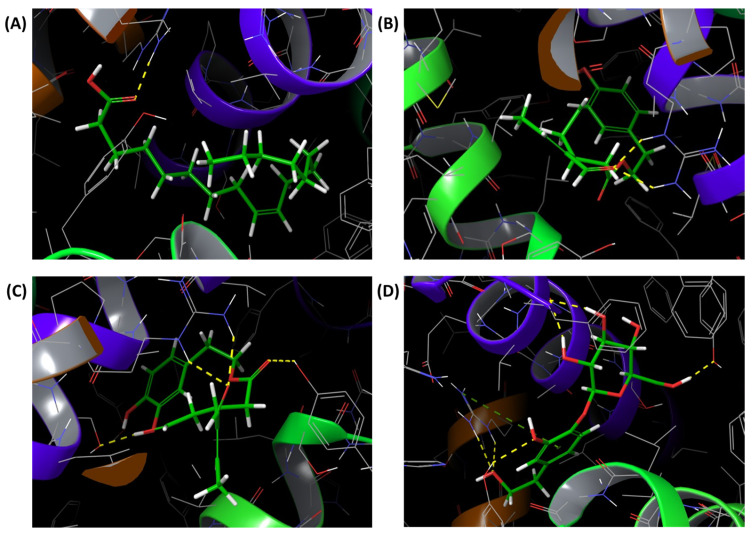
Three-dimensional representation of COX-2 complexed with oleic acid (**A**), oleocanthal (**B**), oleacein (**C**) and hydroxytyrosol 4′-glucuronide (**D**). Yellow dash indicates hydrogen bonds while green dash is related to π–cation interactions.

For both COX isoforms, oleocanthal and oleacein exhibited the most consistent interaction patterns. In addition to π-π interactions, those compounds formed hydrogen-bond networks involving amino-acid residues surrounding the active sites of both COX-1 and COX-2. Again, complexes with hydroxytyrosol 4′-glucuronide were stabilized using hydroxyl groups in a dense network despite the lower ChemPLP values. Conversely, highly hydrophobic compounds such as squalene again displayed high scores but poor interaction profiles (Figure 7 and Figure 8).

## 4. Discussion

Considering the complex etiology of MASLD as well as limited pharmacological options, lifestyle modifications including diet remain the gold standard in the prevention and treatment of this disease [7]. Hence, constituents of diet with multi-target properties may be ideal candidates for MASLD mitigation and management [17,19]. The combination of QSAR-based algorithms, empirical rules, and the molecular docking approach was applied in this research in order to get insights into the drug-likeness properties, potential oral bioavailability as well as interaction mechanisms of multiple active components present in EVOO.

EVOO is rich in lipophilic compounds. The glyceride fraction with up to 85% of unsaturated acid, particularly oleic acid, is the major constituent of olive oil. The less than 2% of olive oil is based on phenolic compounds and vitamin E [20]. Hence, the EVOO compounds diverse in molecular properties were studied in the context of drug-likeness properties and bioavailability. The applied in silico algorithms evaluated absorption and permeability based on passive diffusion processes [21]. According to the predictions conducted by the ADMETlab 3.0 online software tool, poor oral bioavailability was attributed to lipophilic compounds such as squalene, β-sitosterol, vitamin E and oleic acid due to the high logP/logD_7.4_ values. Phenolic compounds also expressed low predicted intestinal absorption due to their high solubility in water. Apart from the molecular descriptors observed in drug-likeness empirical rules (i.e., nRot, nHA, nHD, TPSA, logP), Fsp^3^ is a relatively new parameter considered during the initial phases of drug discovery. The values of 0.42 and higher are usually targeted for compounds with an optimal aqueous solubility and pharmacokinetic profile [22]. Phenolic compounds such as tyrosol, hydroxytyrosol and their sulfate derivatives did not meet the set threshold. While most compounds followed Lipinski’s rule of five and Veber’s rule [15,16], oleuropein exhibited structural constraints (Table 2 and Table 3), suggesting limited passive intestinal permeability and low predicted oral bioavailability. Data from in vitro and in vivo studies confirmed relatively poor and highly variable oleuropein bioavailability [23,24,25]. However, the absorption of EVOO constituents is far more complex. After oral intake, oleuropein is partially hydrolyzed to oleuropein aglycone, hydroxytyrosol and other bioactive metabolites that may also contribute to its biological activity. In addition, hydroxytyrosol forms sulfate and glucuronide conjugates due to the intensive first past metabolism. The bioavailability of phenolic compounds is also gender related and their plasma concentrations are strongly influenced by the type of formulation. For example, liquid forms of oleuropein resulted in better plasma concentrations compared to capsules [26]. The bioavailability of a lipophilic compound is strongly dependent on emulsification with bile salts, the presence of other dietary lipids, and the transport proteins and cellular uptake in the intestinal membrane [23,24]. Taken together, the performed evaluation of molecular descriptors and drug-likeness properties imply that special formulation strategies are required to overcome poor solubility and/or membrane permeability of the studied compounds. Nanoemulsions, liposomes, niosomes, phytosomes as well as polymeric encapsulation systems could offer an alternative approach to enhance the stability and bioavailability of EVOO compounds [27,28]. Considering that poor pharmacokinetic behavior is the most common reason of the failure of promising drug candidates in clinical phases [29], the obtained results might serve as a baseline step for selection of best candidates.

Combined molecular docking and binding-mode analyses supported the hypothesis that EVOO-derived compounds may have acted as multi-target modulators of MASLD-related pathways (Table 4). Molecular targets involved in disease pathogenesis were carefully chosen based on their involvement in disease onset and progression as well as the activity of their co-crystalized ligands. For example, T0901317, lanifibranor, and GW1929 act as agonists for LXRα/β and PPARα/γ, respectively [29,30,31], while mevastatin, flurbiprofen and naproxen behave as inhibitors of HMG-CoA reductase, COX-1 and COX-2, respectively [32,33,34]. Despite the still-debated role of LXR in MASLD, for more than a decade, LXR α/β agonists have been considered for the treatment of dyslipidemia and atherosclerosis [35]. Binding-mode analysis demonstrated that phenolic compounds present in EVOO are able to interfere with key amino acids within the LXRα binding cavity (His421), specifically oleuropein, tyrosol, hydroxytyrosol, and its glucuronide and sulfate derivatives. Moreover, tyrosol, hydroxytyrosol, and hydroxytyrosol 3′-sulfate derivatives, like endogenous agonists oxycholesterols, established an additional hydrogen bond with Trp443 [36]. A novel in vivo study highlighted that the intestinal activation of LXRα resulted in decreased inflammation, steatosis, and liver fibrosis in MASLD [37]. Interestingly, the most abundant phytosterol in olive oil, β-sitosterol, reproduced a hydrogen bond with His435 as proven LXRβ agonist, T0901317. Despite lower ChemPLP values, hydroxytyrosol 4′-glucuronide and hydroxytyrosol 3′-sulfate reassembled the same binding mode of interaction while oleuropein, vitamin E and oleocanthal interacted via hydrogen bonds with Thr272, a residue reported to participate in ligand recognition within the LXRβ binding pocket [38]. Oleuropein aglycon and oleocanthal also had hydrophobic interactions involving Phe329 at the binding site, similar to other ligands for therapeutic utility [39].

PPARα/γ are primary targets in MASLD treatments due to their important role in lipid metabolism, insulin sensitivity, and inflammation [40]. Fibrates, like the co-crystallized ligand, lanifibranor, promote HDL formation and decrease triglyceride levels in blood via PPARα receptor activation [41]. The increased stability of phenolic compounds inside the binding pocket was attributed to the multiple hydrogen bonds. Previous in silico studies of PPARα agonists highlighted the role of hydrogen bonds with Ser280 and His440 amino-acid residues [42]. Oleacein and hydroxytyrosol 4′-glucuronide established hydrogen bonds with Ser280 analogous to fibrates, while oleuropein and hydroxytyrosol 4′-glucuronide interacted with the receptor, creating hydrogen bonds with His440 [42,43]. In the literature, the critical role of Phe273 in ligand-binding selectivity, stability and PPARα conformation is firmly established [44]. Hence, hydrogen bonds involving this residue additionally increased the stability of oleuropein, vitamin E, and oleocanthal within the bonding cavity. Targeting PPARγ represents a well-known approach in MASLD treatment, due to its ability to improve insulin sensitivity and reduce blood glucose levels [17]. Multiple hydrogen bonds contributed to the stability of lipophilic and hydrophilic EVOO compounds within the binding cavity. PPARγ analysis showed a hydrogen-bond binding mode involving hydrophilic residues His323, Tyr327, Lys367, His449, similar to the typical agonist rosiglitazone. In addition, bonding with Phe282 within hydrophobic region was also observed [45]. Despite the lower ChemPLP scores, 3′-hydroxytyrosol 3′-glucuronide, hydroxytyrosol 4′-glucuronide, oleuropein aglycon and oleocanthal established multiple hydrogen interactions.

Despite a range adverse effects, statins, as HMG-CoA reductase inhibitors, are commonly administered to MASLD patients. The inhibition of this enzyme is a primary therapeutic strategy for the reduction in cardiovascular risk as well as for the prevention of hepatic steatosis and fibrosis [46]. A dense network of interactions with key residues common for statins Arg590, Asp690, Lys691, Lys735 was observed in the case of oleuropein [47]. Vitamin E also formed hydrogen bonds with Arg590 and Lys691. Oleacein and oleuropein aglycone stability were increased via bonding with Arg590, Lys735 and Ala751. Importantly, oleacein was the only compound that established hydrogen bond with key residue Ser684, similar to statins [17,47].

Keeping in mind the pro-inflammatory role of prostaglandins in MASLD, COX-1 and COX-2 were examined as potential targets for EVOO compounds. Docking analysis suggested that these compounds might mitigate hepatic inflammation, lipid accumulation, and progression of the disease through the combination of hydrogen and π-π interactions with residues located within the enzyme’s binding site. The active sites of both isoforms are created of almost the same amino-acid residues, and hydrogen-bond interactions with Arg120 and Tyr355 have been reported as characteristic features of the binding modes of many COX inhibitors [48]. Based on the target-specific binding-mode analysis, oleocanthal, oleacein, vitamin E, oleic acid reassembled the hydrogen bond with Arg120 while hydroxytyrosol 4′-glucuronide, despite the lower ChemPLP values, established additional hydrogen bond with Tyr355.

Although lacking experimental validation and molecular dynamics simulations, this in silico study provides an initial insight into the drug-likeness properties, binding affinities, and interaction profiles of EVOO-derived compounds against key targets involved in MASLD pathogenesis. PPARα and PPARγ are currently among the most extensively investigated pharmacological targets, with compounds such as co-crystallized lanifibranor showing promising results in clinical studies. LXR receptors remain experimental targets but play a central role in the regulation of lipid and cholesterol metabolism. HMG-CoA reductase is the well-established target of statins, drugs widely used in patients with MASLD because of their cardiometabolic benefits. In addition, COX-mediated pathways contribute to hepatic and systemic inflammation, suggesting that the modulation of these enzymes may represent an additional mechanism through which EVOO-derived compounds exert beneficial effects in MASLD. Based on these findings oleuropein, oleocanthal, and oleacein should be considered in the management of MASLD due to the exhibited moderate to high potential for a multi-target approach (Table 5). However, further molecular dynamics simulations and experimental assays should provide additional physiologically relevant assessments of compound and target engagement.

One of the major limitations of the performed docking analysis is the application of scoring functions for the evaluation of binding affinities. ChemPLP has been widely used as the default scoring function in GOLD due to the highest success rates (for both pose prediction and virtual screening experiments) in comparison with diverse validation test sets [49]. However, the obtained high ChemPLP scores are not always associated with biologically relevant binding modes. The score is calculated based on an empirical approach that combines van der Waals interactions, hydrogen bonding and electrostatic interactions [50]. Hydrophobic compounds with high logP values such as squalene, vitamin E, β-sitosterol and oleic acid, frequently exhibited elevated scores despite lacking stabilizing interactions within the ligand-binding domain. Hence, the high hydrophobicity of these compounds should not be neglected in the discussion of the obtained ChemPLP scores. As stated in this research, the binding sites of the co-crystallized ligands were used to significantly increase the docking efficiency. Consequently, ChemPLP scores could only be used in comparison with the score obtained for the co-crystallized ligand for each target and could not be directly compared across targets with different pocket properties. In addition, the molecular docking results were discussed based on the binding profiles and established interactions within the ligand-binding pocket. A limitation of the present docking protocol is that all crystallographic water molecules were removed during target preparation to ensure methodological consistency across all targets. This approach neglected the structurally conserved water molecules that could mediate water-bridged hydrogen-bond interactions, particularly within the COX-2 active site and the ligand-binding domains of PPAR receptors. Although interactions with certain amino-acid residues have been reported for recognized agonists or inhibitors, similar behaviors may also occur in complexes with ligands exhibiting different functional profiles. Therefore, the observed binding modes should not be interpreted as evidence of agonistic or inhibitory activity without further in vitro and in vivo experiments. In addition, the relatively low predicted oral bioavailability of oleuropein might be overcome with advances in drug delivery technologies and pharmacokinetic evidences about its metabolism to oleuropein aglycone and hydroxytyrosol, which may also contribute to its biological activity. Hence, both the molecules and their metabolites were also tested using docking analysis. But the molecular species interacting with the target receptors in vivo may differ from the tested compounds due to gastrointestinal and metabolic conversion.

From a clinical perspective, the present findings support the growing body of evidence suggesting that EVOO should be considered not only as a dietary component but also as a source of bioactive molecules potentially capable of modulating multiple pathogenic pathways involved in MASLD. The multi-target interactions identified for oleuropein, oleocanthal, and oleacein are particularly relevant, as MASLD is characterized by a complex interplay between liver steatosis, insulin resistance, chronic inflammation, dyslipidemia, and cardiovascular risk [5,51]. The simultaneous modulation of PPARα/γ signaling, cholesterol metabolism, inflammatory pathways, and lipid homeostasis may provide a mechanistic explanation for the beneficial effects consistently observed in the Mediterranean diet intervention [6]. Therefore, therapeutic strategies targeting a single molecular pathway often provide only partial benefits. In this context, EVOO-derived compounds may represent attractive adjunctive agents capable of exerting complementary effects on both hepatic and extrahepatic manifestations of the disease. Although the present results are limited to in silico predictions and cannot be directly translated into clinical recommendations, they provide a biologically plausible rationale for further experimental and clinical investigations.

Future studies should focus on validating these molecular interactions in cellular and animal models, assessing the bioavailability of individual EVOO phenolics in optimized formulations, and determining whether specific compounds or combinations can enhance the therapeutic effects of lifestyle interventions. Ultimately, a better understanding of the pharmacological potential of EVOO-derived molecules may contribute to the development of novel nutraceutical or adjunctive therapeutic approaches for MASLD, particularly in patients with high cardiometabolic risk.

## 5. Conclusions

This integrated in silico approach, combining molecular docking, drug-likeness evaluation, and binding-mode analysis, identified several EVOO-derived bioactive compounds with potential relevance for MASLD management. Among the investigated molecules, oleuropein, oleacein, and oleocanthal emerged as the most promising multi-target candidates, showing consistent interaction profiles across molecular pathways involved in lipid metabolism, inflammation, and cardiometabolic regulation. Importantly, the combined assessment of docking scores and binding interactions highlighted the need for cautious interpretation of highly lipophilic compounds, whose elevated docking scores may not necessarily reflect biologically meaningful target engagement. Although oleuropein exhibited the most favorable overall interaction profile, its predicted pharmacokinetic limitations suggest that bioavailability and metabolic transformation should be carefully considered when translating these findings into biological settings. Overall, the present study provides a mechanistic framework supporting the beneficial effects of EVOO within the Mediterranean diet and identifies promising candidate molecules for future translational and experimental studies in MASLD.

## Figures and Tables

**Table 1:** Selected targets with co-crystallized ligands.

Target	PDB Code	Co-Crystalized Ligand
LXRα	1UHL	T0901317
LXRβ	1UPV	T0901317
PPARα	8HUK	lanifibranor
PPARγ	6D8X	GW1929
HMG-CoA reductase	1HW8	mevastatin
COX-1	3N8Z	flurbiprofen
COX-2	3NT1	naproxen

Abbreviations: PDB, Protein Data Bank; LXRα, Liver X Receptor alpha; LXRβ, Liver X Receptor beta; PPARα, Peroxisome Proliferator-Activated Receptor alpha; PPARγ, Peroxisome Proliferator-Activated Receptor gamma; HMG-CoA reductase, 3-Hydroxy-3-Methylglutaryl-Coenzyme A Reductase; COX-1, Cyclooxygenase-1; COX-2, Cyclooxygenase-2.

**Table 2:** Molecular properties of analyzed compounds obtained by ADMETlab 3.0.

Ligand/Compound	M_W_	V	nRing	nRot	nHA	nHD	TPSA	Fsp^3^
3′-Hydroxytyrosol 3′-Glucuronide	316.12	296.0	2	5	8	6	139.84	0.571
β-Sitosterol	414.39	482.068	4	6	1	1	20.23	0.931
Homovanillic Acid	182.06	180.279	1	3	4	2	66.76	0.222
Hydroxytyrosol 4′-glucuronide	330.1	302.154	2	5	9	6	156.91	0.5
Hydroxytyrosol	154.06	156.829	1	2	3	3	60.69	0.25
Hydroxytyrosol 3′-sulfate	234.02	201.709	1	4	6	3	104.06	0.25
Oleacein	320.13	328.318	1	10	6	2	100.9	0.353
Oleanolic Acid	456.36	505.751	5	1	3	2	57.53	0.9
Oleic Acid	282.26	332.192	0	15	2	1	37.3	0.833
Oleocanthal	304.13	319.528	1	10	5	1	80.67	0.353
Oleuropein aglycone	378.13	371.934	2	8	8	3	122.52	0.368
Oleuropein	540.18	511.104	3	11	13	6	201.67	0.52
Squalene	410.39	511.617	0	15	0	0	0	0.6
Tyrosol 4-sulfate	218.02	192.919	1	4	5	2	83.83	0.25
Tyrosol	138.07	148.039	1	2	2	2	40.46	0.25
Vitamin E	430	502.698	2	12	2	1	29.46	0.793

Abbreviations: M_W_, molecular weight; V, volume; nRing, number of rings; nRot, number of rotatable bonds; nHA, number of hydrogen-bond acceptors; nHD, number of hydrogen-bond donors; TPSA, topological polar surface area; Fsp^3^, fraction of sp^3^ carbons.

**Table 3:** Lipophilicity, water solubility, intestinal absorption, and blood–brain barrier permeability parameters of the analyzed compounds.

Ligand/Compound	logP	LogD_7.4_	LogS	HIA	BBB
3′-Hydroxytyrosol 3′-Glucuronide	−0.853	−0.277	−0.852	+	−
β-Sitosterol	8.004	5.37	−7.221	−	−
Homovanillic Acid	0.809	0.873	−1.011	−	−
Hydroxytyrosol 4′-glucuronide	−1.034	−0.357	−0.536	−	−
Hydroxytyrosol	0.453	0.512	−0.566	−	−
Hydroxytyrosol 3′-sulfate	−0.573	0.482	−1.329	+	−
Oleacein	1.946	1.848	−2.656	−	−
Oleanolic Acid	4.11	3.544	−5.061	−	+
Oleic Acid	7.063	3.703	−5.865	−	−
Oleocanthal	2.005	1.908	−2.945	−	−
Oleuropein aglycone	2.242	2.305	−3.266	−	−
Oleuropein	0.743	1.157	−2.037	−	−
Squalene	11.169	5.744	−10.531	−	−
Tyrosol 4-sulfate	−0.164	0.724	−1.271	+	−
Tyrosol	0.582	0.589	−0.761	−	−
Vitamin E	9.477	5.539	−8.105	−	+

Abbreviations: logS, the logarithm of aqueous solubility value; logP, the logarithm of the n-octanol/water distribution coefficient; logD_7.4_, the distribution coefficient at physiological pH; HIA, human intestinal absorption; BBB, blood–brain barrier penetration.

**Table 4:** ChemPLP fitness score obtained from molecular docking of selected compounds towards selected targets in comparison to co-crystalized ligands.

Ligand/Compound	LXRα	LXRβ	PPARα	PPARγ	HMG-CoA Reductase	COX-1	COX-2
T0901317	68.541	82.778	/	/	/	/	/
lanifibranor	/	/	91.781	/	/	/	/
GW1929	/	/	/	113.039	/	/	/
mevastatin	/	/	/	/	81.809	/	/
flurbiprofen	/	/	/	/	/	78.661	/
naproxen	/	/	/	/	/	/	74.702
3′-Hydroxytyrosol 3′-Glucuronide	59.882	50.804	49.579	61.161	55.778	50.978	50.045
β-Sitosterol	75.323	76.922	64.491	70.784	50.637	36.077	47.434
Homovanillic Acid	38.916	48.069	44.148	52.611	40.975	46.415	46.675
Hydroxytyrosol 4′-glucuronide	56.430	55.138	61.100	68.445	56.453	60.814	64.239
Hydroxytyrosol	44.700	55.138	45.964	48.744	45.875	45.392	43.227
Hydroxytyrosol 3′-sulfate	49.492	42.254	48.193	53.619	47.048	49.084	51.749
Oleacein	64.050	62.129	65.235	76.867	60.585	72.746	74.332
Oleanolic Acid	68.482	44.991	38.874	19.978	45.630	/	/
Oleic Acid	66.066	72.510	73.721	77.639	60.017	68.099	76.573
Oleocanthal	62.298	65.929	60.381	73.080	56.428	73.426	76.293
Oleuropein aglycone	61.637	66.629	69.482	71.561	60.598	61.590	61.464
Oleuropein	84.216	89.037	80.186	72.927	69.826	19.460	37.828
Squalene	86.936	92.279	98.227	113.317	72.882	76.629	89.432
Tyrosol 4-sulfate	41.659	45.686	48.860	52.393	44.361	50.291	51.473
Tyrosol	40.024	41.409	48.945	49.638	39.062	45.745	44.215
Vitamin E	80.794	86.328	84.155	103.524	65.750	72.068	60.829

“/”—docking was not converged. Abbreviations: LXRα, Liver X Receptor alpha; LXRβ, Liver X Receptor beta; PPARα, Peroxisome Proliferator-Activated Receptor alpha; PPARγ, Peroxisome Proliferator-Activated Receptor gamma; HMG-CoA reductase, 3-Hydroxy-3-Methylglutaryl-Coenzyme A Reductase; COX-1, Cyclooxygenase-1; COX-2, Cyclooxygenase-2.

**Table 5:** Overall potential of analyzed compounds.

Compound	Drug-Likeness	Multi-Target Activity	Overall Potential in MASLD
Oleuropein	Low	High	Moderate
Oleocanthal	Moderate	Moderate to high	High
Oleacein	Moderate	Moderate to high	High
Hydroxytyrosol derivatives	High	Moderate	Moderate
Vitamin E	Low	Moderate	Moderate
Squalene	Low	Low	Low

The overall potential was evaluated using qualitative decision-making matrix that balances multi-target activity and drug-likeness properties; High—ChemPLP scores, relevant binding mode and adherence to drug-likeness rules; Moderate to high—certain binding modes require further optimization; Moderate—violation in up to two drug-likeness criteria or lower ChemPLP scores despite observed interactions; Low—violation of drug-likeness rules and absence of relevant interactions; Abbreviations: MASLD, Metabolic Dysfunction-Associated Steatotic Liver Disease.

## Data Availability

The original contributions presented in this study are included in the article. Further inquiries can be directed to the corresponding author.

## Abbreviations

The following abbreviations are used in this manuscript:

BBB	Blood–Brain Barrier
COX-1	Cyclooxygenase-1
COX-2	Cyclooxygenase-2
EVOO	Extra Virgin Olive Oil
Fsp^3^	Fraction of sp^3^ Carbon Atoms
GOLD	Genetic Optimisation for Ligand Docking
HIA	Human Intestinal Absorption
HMG-CoA Reductase	3-Hydroxy-3-Methylglutaryl-Coenzyme A Reductase
LXRα	Liver X Receptor Alpha
LXRβ	Liver X Receptor Beta
MASLD	Metabolic Dysfunction-Associated Steatotic Liver Disease
MW	Molecular Weight
nHA	Number of Hydrogen-Bond Acceptors
nHD	Number of Hydrogen-Bond Donors
nRing	Number of Rings
nRot	Number of Rotatable Bonds
PDB	Protein Data Bank
PPARα	Peroxisome Proliferator-Activated Receptor Alpha
PPARγ	Peroxisome Proliferator-Activated Receptor Gamma
RMSD	Root Mean Square Deviation
TPSA	Topological Polar Surface Area
V	Volume
